# Epidemiology and phenomenology of the Charles Bonnet syndrome in low-vision patients

**DOI:** 10.1007/s10792-024-03298-0

**Published:** 2024-09-10

**Authors:** Sophia E. G. Christoph, Karl T. Boden, Annette Pütz, Kai Januschowski, Rudolf Siegel, Berthold Seitz, Peter Szurman, André Schulz

**Affiliations:** 1 Eye Clinic Sulzbach, Knappschaft Hospital Saar, Sulzbach/Saar, Germany; 2 Klaus Heimann Eye Research Institute (KHERI), Sulzbach/Saar, Germany; 3 Department of Psychology, Saarland University, Saarbrücken, Germany; 4 Department of Ophthalmology, Saarland University Medical Center UKS, Homburg, Germany; 5 Present Address: Mount Saint Peter Eye Clinic, Trier, Germany; 6 Present Address: Department of Ophthalmology, Rostock University Medical Center, Doberaner Str. 140, 18057 Rostock, Germany

**Keywords:** Visual hallucinations, Charles Bonnet’s Syndrome, Prevalence, Low-vision patients, Macular degeneration, Epidemiology

## Abstract

**Background:**

The occurrence of visual hallucinations in visually impaired people without mental impairment is known as Charles Bonnet Syndrome (CBS). To date, the prevalence of CBS has been reported with high variance. The present study aims at evaluating the prevalence of CBS among low-vision patients.

**Methods:**

From March 2018 to February 2022, 194 patients with a visual acuity ≥ 0.5 logMAR approached the low vision section of the Eye Clinic Sulzbach. Of these, 50 patients were found eligible, agreed to participate in the study and were screened for CBS. The course of the disease, its phenomenology and characteristics, the circumstance of onset, the ability to manipulate and resolve the hallucinations, and the psychosocial aspects of CBS were investigated.

**Results:**

26% of patients with low vision suffered from CBS. Women did not suffer from CBS significantly more often than men. Often, insight into the unreality of the images is not achieved immediately. Patterns or so-called “simple” hallucinations occurred just as frequently as other types of images such as people, body parts or faces. The most frequent images were animals. Visual hallucinations, lasting only for seconds in most cases, occurred more frequently during the day and in bright surroundings. All patients experienced the hallucinations exclusively with their eyes open. The hallucinations generally did not move with the eyes. Many sufferers did neither communicate about their hallucinations nor consult any physician.

**Conclusions:**

CBS among low-vision patients is common. Its prevalence constitutes clinical relevance. Future management of CBS may benefit from encouraging patients to share their experiences and consult a physician.

**Supplementary Information:**

The online version contains supplementary material available at 10.1007/s10792-024-03298-0.

## Introduction

Visual hallucinations (VH) in a low sighted individual were first described by Charles Bonnet in 1769 [1]. Later, George DeMorsier coined the term Charles Bonnet’s Syndrome (CBS) [2]. It is characterised by visual hallucinations in visual impaired, mentally healthy individuals who generally are aware of the unreality of the images. Diagnostic criteria were proposed in 1989 by Gold and Rabins [3], based on a suggestion made in 1982 by Damas-Mora et al. [4]. Variants of these two sets of criteria have since been used most frequently. Accordingly, CBS is characterised by visual hallucinations that are (1) formed, (2) complex and (3) persistent or repetitive and (4) stereotyped. In general, patients have full or partial insight in the unreality of these images. Also, primary or secondary delusions or hallucinatory phenomena in other sensory modalities are usually not present, though “CBS plus” (e.g. CBS hallucinations with matching sound) has been described [5, 6]. While there are some systematic studies on patient symptoms, most knowledge about CBS is based on case reports and anecdotes. The prevalence of CBS reported in literature varies between 0.47% among all ophthalmologic patients [7] and 40% among patients with age-related macular degeneration (AMD) [8].

Low vision has been identified as one of several risk factors for CBS [9, 10], as well as bilateral vision loss [11] and advanced age [9, 12]. Additionally, the intake of proton pump inhibitors is also currently being discussed as a risk factor for CBS [13, 14]. Findings about social isolation as a risk factor have been contradictory [7, 15, 16]. Whilst differences in sex distribution were also noticed, it remains unclear whether sex might be a risk factor for CBS [4, 15]. To date, a variety of studies have investigated the prevalence of CBS among low-vision patients [7, 9, 11, 17–24] ranging from 5.7[17]–34% [11]. Here, the prevalence was mostly reported as less than 20%. Only one study found a higher prevalence of CBS (34%) in patients with a best corrected visual acuity (BCVA) of ≥ 0.30 logMAR [11].

Therefore, the present study aims to provide additional data to further assess the prevalence of CBS in the population of low-vision patients and to examine the characteristics of the visual hallucinations experienced by CBS-patients, especially regarding sex distribution, insight in the hallucinations, their content, occurrence, the ability of arbitrary manipulation/resolution and their psychosocial impact.

## Methods

### Study design and subjects

A cross-sectional, prospective study was conducted. Between March 2018 and February 2022, 194 patients attending the low vision department at the Eye Clinic Sulzbach were informed about the study. After having given consent, adult patients (≥ 18 years old) with low vision were included. Low vision was defined as a visual acuity (VA) ≥ 0.5 logMAR, according to the ICD-11 criteria for vision impairment [25]. Basic demographic data like age, sex, VA and ophthalmic diagnoses were collected. Best-corrected VA was assessed using ETDRS charts and presented as the logarithmic minimum angle of resolution (logMAR). Then, patients were asked a screening question for CBS: “Some people with low visual acuity reported seeing things that are not there. Has it ever happened to you that you saw similar optical hallucinations?” Those who answered with yes underwent further interviewing, which was carried out according to a standardized questionnaire adapted from Teunisse et al. [20] containing 27 questions about information on phenomenology and characteristics of the hallucinations, length of symptoms, the report of them and knowledge about the syndrome among physicians (ref. Supplementary Information).

194 patients attended the low vision department at the Eye Clinic Sulzbach. Of those, 50 patients met all inclusion criteria (written, informed consent, age over 18 years old, VA ≥ 0.5 logMAR) and therefore were included in the present study. A diagnosis of CBS was assigned when individuals reported visual hallucinations that could not be explained by entoptic phenomena (floaters, retinal detachment) regardless of whether they were simple or complex. As the patient’s written, informed consent was a prerequisite for participating in the study, patients with cognitive impairment (e.g., Alzheimer’s dementia) were not included. Other diseases that could cause visual hallucinations (e.g., hypnagogic/hypnopompic hallucinations or major psychiatric disorders like schizophrenia, alcohol abuse or delirium) as well as the intake of psychoactive substances and hallucinogenic medication were also excluded by careful anamnesis and interview.

The study was conducted in accordance with the ethical standards of the Helsinki Declaration, was registered in the German Clinical Trials Register (No. DRKS00021065) and approved by the local ethics committee (No. 256/17). Participation in the study was voluntary. Consent could be withdrawn at any time without giving reasons. Study participants were informed verbally and in writing about the nature and scope of the planned study before its start. No included patient withdrew from the study.

### Statistical evaluation

Data analysis was performed using R (version 4.1). A graphical illustration of the data was performed using OriginPro software. The data are presented as mean ± standard deviation. Differences between groups were tested using t-test, Chi-squared tests and Pearson correlations. Statistical tests were considered significant if *p* < 0.05.

## Results

### Prevalence of CBS in patients with low vision

50 patients were included with an average age of 75.2 ± 9.49 years (range 55–85 years) (Table 1). Out of 50 respondents, 13 patients suffered from CBS resulting in a prevalence of 26.0% for CBS in patients with low vision. Both age (Δ*M* = 5.16, 95% CI [−1.35, 11.67], *p* = 0.114) and mean best corrected visual acuity (BCVA) of the right (Δ*M* = −0.09, 95% CI [−0.46, 0.27], *p* = 0.611) nor left eye (Δ*M* = −0.03, 95% CI [−0.44, 0.38], *p* = 0.876) did not differ significantly between CBS and non-CBS patients. However, a higher proportion of females in the CBS group in comparison to the non-CBS group was found, yet without statistical significance (*χ*2(1,*N* = 50) = 1.64, *p* = 0.200).

**Table 1:** Clinical characteristics of patients

Variable	CBS patients (*n* = 13, 26.0%)	Non-CBS patients (*n* = 37, 74.0%)	Overall (*N* = 50, 100%)
Age in years (mean ± SD)	71.4 ± 9.82	76.6 ± 9.13	75.2 ± 9.49
VA right eye in logMAR (mean ± SD)	1.16 ± 0.53	1.07 ± 0.59	1.09 ± 0.57
VA left eye in logMAR (mean ± SD)	1.28 ± 0.61	1.25 ± 0.61	1.25 ± 0.60
Ratio female to male, F:M	10:3	19:18	29:21

*SD* Standard Deviation, *VA* visual acuity, F:M female to male ratio

**Fig. 1:**
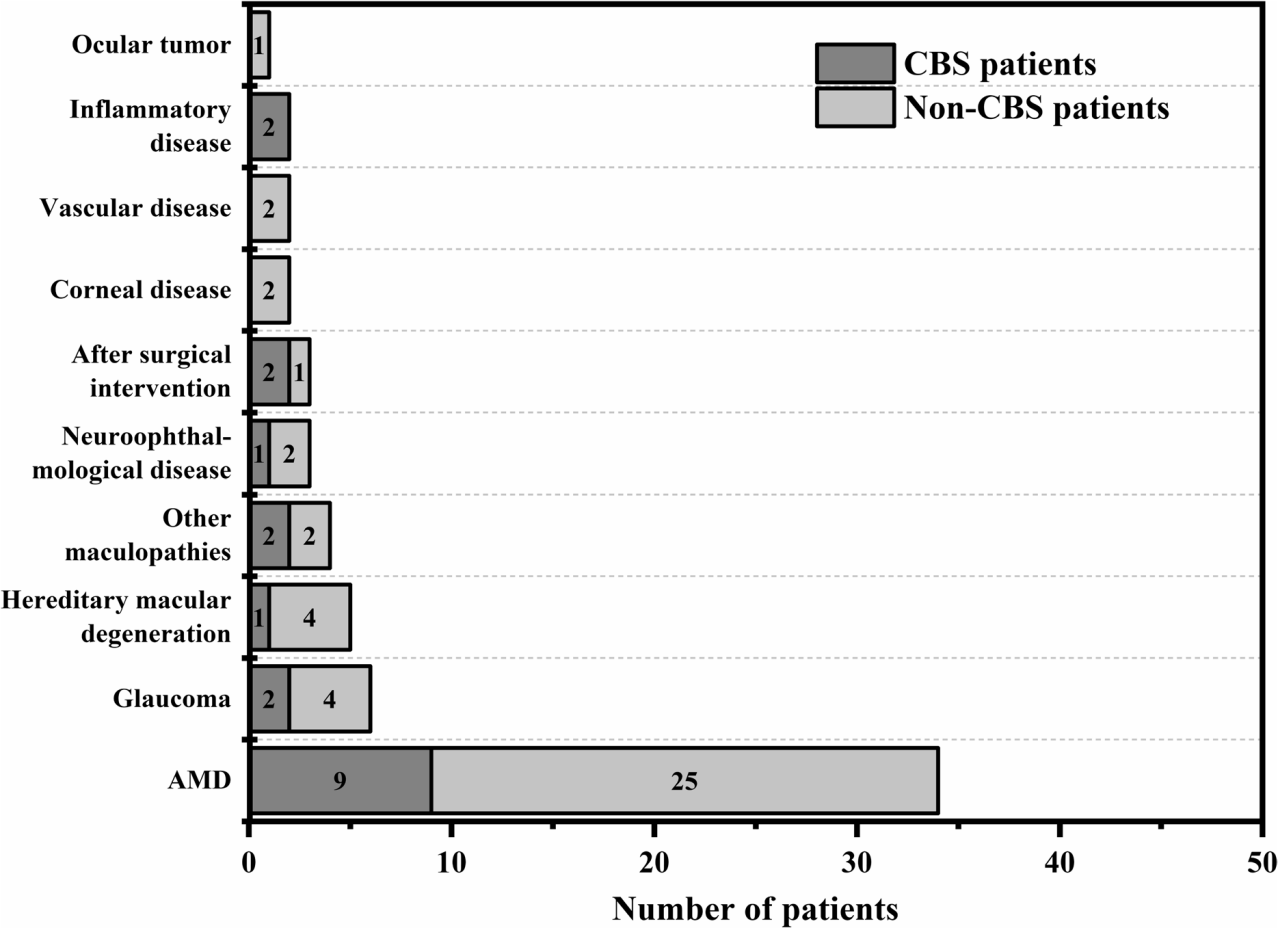
Graphic illustration of the distribution of ophthalmic conditions among the overall population. Multiple selections were possible

With regard to the ophthalmic conditions from which CBS patients suffered, AMD was found to be the most common cause of visual impairment (Fig. 1; Table 2). Patients often suffered from several ophthalmic conditions at the same time, e.g., AMD and glaucoma. The most frequent general comorbidities were musculoskeletal, accounting for 6 out of 13 patients in this group. Other common diagnoses suffered by CBS patients were arterial hypertension, diabetes and cardiovascular diseases (e.g., cardiac insufficiency, atrial fibrillation or condition after stroke). One patient was found to have been taking antipsychotics, but these had only been administered after the onset of hallucinations in a short, unsuccessful treatment attempt. Therefore, the patient was not excluded from the study.

**Table 2:** Pre-existing general and ophthalmic conditions of CBS patients (*n* = 13)

General medical conditionS	% (N)	Ophthalmic conditions	% (N)
Musculoskeletal	46.2 (6/13)	AMD	69.2 (9/13)
Cardiovascular disease	30.8 (4/13)	Glaucoma	15.4 (2/13)
Diabetes	23.1 (3/13)	Myopic maculopathy	15.4 (2/13)
Arterial hypertension	23.1 (3/13)	Keratoplasty	7.69 (1/13)
Immunologic-inflammatory history	15.4 (2/13)	Scleral buckle surgery	7.69 (1/13)
Hypothyroidism	15.4 (2/13)	Intermediate uveitis	7.69 (1/13)
Gastrointestinal disease	15.4 (2/13)	Occlusive vasculitis of unknown etiology	7.69 (1/13)
Sensory impairment other than vision	7.69 (1/13)	Amblyopia	7.69 (1/13)
benign prostatic hyperplasia	7.69 (1/13)	Hereditary macular degeneration	7.69 (1/13)

Multiple selections were possible

*CBS* Charles Bonnet’s Syndrome, *AMD* age-related macular degeneration

### Characteristics of the visual hallucinations

Table 3 summarises the characteristics of visual hallucinations described by low-vision patients with CBS. Most patients had been suffering from visual hallucinations for more than a year at the time they were interviewed. Some had been seeing them for only a short time, i.e. days, weeks or months, and one patient had been living with the hallucinations for over ten years. The hallucinations occurred with varying frequencies, several times a day or less frequently than monthly. There was a slight tendency for hallucinations to occur once or more per week. Although one patient suffered from constant hallucinations, in most cases the images only occured for seconds (84.6%). Insight into the unreality of the images was not always given from beginning. Almost half of the patients were not aware that the images they saw were unreal, the same number of patients recognised the unreality of the VH at first sight.

**Table 3:** Characteristics of the visual hallucinations

Characteristic	% (N)	Characteristic	% (N)
*First time of occurrence*		*Frequency*	
2 weeks ago	7.69 (1/13)	Multiple times per day	7.69 (1/13)
> 2 weeks, < 4 weeks ago	23.1 (3/13)	Daily	7.69 (1/13)
> 1 month, < 6 months ago	7.69 (1/13)	Multiple times per week	23.1 (3/13)
> 6 months, < 1 year ago	15.4 (2/13)	Weekly	23.1 (3/13)
> 1 year ago	38.5 (5/13)	Monthly or less	7.69 (1/13)
> 10 years ago	7.69 (1/13)	Sporadically	23.1 (3/13)
		Special situation	7.69 (1/13)
*Duration*		*Content*	
Seconds	84.6 (11/13)	Animals, animalic creatures, monsters	61.5 (8/13)
Days	7.69 (1/13)	Patterns, shapes, stains, colours, caleidoscopic	30.8 (4/13)
Continuously	7.69 (1/13)	People, figures, children, bodyparts	30.8 (4/13)
		Inanimate objects	23.1 (3/13)
Insight in unreality from onset		Plants, flowers, trees	7.69 (1/13)
No	46.2 (6/13)	Dwarfs, giants, humanoid creatures	7.69 (1/13)
Yes	46.2 (6/13)	Warped surroundings	7.69 (1/13)
NA	7.69 (1/13)		
*Chromaticity*		*Matching the surrounding*	
Black and white	46.2 (6/13)	No	38.5 (5/13)
Coloured/colourful	30.8 (4/13)	Yes	46.2 (6/13)
Specific colour	15.4 (2/13)	Partially	7.69 (1/13)
Unknown/NA	7.69 (1/13)	NA	7.69 (1/13)
*Moving with eyes*		*Clarity compared to patient’s normal sight*	
No	61.5 (8/13)	Clearer images	30.8 (4/13)
Yes	7.69 (1/13)	Less clear images	46.2 (6/13)
Intrinsic movement	7.69 (1/13)	Equally clear	7.69 (1/13)
Unknown/NA	23.1 (3/13)	Varying	15.4 (2/13)
*Intrinsic movement*		*Repetitiveness*	
No	15.4 (2/13)	No	0.00 (0/13)
Yes	84.6 (11/13)	Yes	100 (13/13)
*Occurrence with eyes open/closed*		*Familiarity of content*	
Eyes open	100 (13/13)	No	30.8 (4/13)
Eyes closed	0.00 (0/13)	Yes	53.8 (7/13)
		Partially familiar	15.4 (2/13)
*Specific time of day*		*Occurrence in special situations*	
Daytime, not further specified	46.2 (6/13)	No special situation of occurrence	69.2 (9/13)
Morning	7.69 (1/13)	When tired	7.69 (1/13)
Afternoon	7.69 (1/13)	When active	7.69 (1/13)
Evening/at dawn	15.4 (2/13)	At night, when waking up	7.69 (1/13)
Daytime and nighttime	7.69 (1/13)	Other	7.69 (1/13)
Always	7.69 (1/13)		
NA	7.69 (1/13)		
*Luminosity of surrounding*		*Whereabouts at occurrence*	
Bright surrounding, good lighting	46.2 (6/13)	At home	38.5 (5/13)
Bad lighting	15.4 (2/13)	Outside	15.4 (2/13)
Dim light	7.69 (1/13)	Both, at home and outside	23.1 (3/13)
Indifferent	23.1 (3/13)	Special situation	15.4 (2/13)
NA	7.69 (1/13)	NA	7.69 (1/13)
*Social surrounding at occurrence*			
Alone	38.5 (5/13)		
In company	30.8 (4/13)		
Both	30.8 (4/13)		

Characteristic	% (N)	Characteristic	% (N)
*Intentional manipulation*		*Sensations when content appears*	
Not possible	38.5 (5/13)	Fear	15.4 (2/13)
Make VH disappear spontaneously	15.4 (2/13)	Fear, only in the beginning	15.4 (2/13)
Manipulation by eye movements	7.69 (1/13)	Feeling uneasy	7.69 (1/13)
Manipulation by blinking	7.69 (1/13)	Annoying	7.69 (1/13)
Manipulation by closing the eyes	7.69 (1/13)	Varying	7.69 (1/13)
Didn’t try to manipulate images yet	7.69 (1/13)	No fear or indifference	53.8 (7/13)
NA	15.4 (2/13)		
*Disappearance of the content*		*Communication of the VH*	
Not possible	30.8 (4/13)	No	38.5 (5/13)
Disappear spontaneously	30.8 (4/13)	Generally yes	23.1 (3/13)
Blinking	7.69 (1/13)	Spoken with family	30.8 (4/13)
Close eyes	7.69 (1/13)	Spoken with friends/acquaintances	7.69 (1/13)
NA	23.1 (3/13)		
*Apprehension VH originate from other condition*		*Consultation of physician due to VH*	
Yes	23.1 (3/13)	No consultation so far	76.9 (10/13)
No	69.2 (9/13)	Consultation of ophthalmologist	15.4 (2/13)
NA	7.69 (1/13)	Consultation of neurologist	7.69 (1/13)

Patients were asked to describe what they were hallucinating. In most cases (61.5%) patients saw animals, animal-like creatures or monsters, with cats appearing quite frequently (6/13 mentioned cats). Animals were followed by humans (30.8%) and abstract images (30.8%). Other images included inanimate objects, plants and dwarves. One person saw their surroundings distorted. Regarding the circumstances of the occurrence of the VH, it was found that hallucinations exclusively occurred when the patients had their eyes open (13/13). The images appeared at different times throughout the day, mostly during the daytime with a slight tendency towards the evening. Although one might expect the VH to occur in a dimmer environment, patients reported seeing them mainly in a bright environment with good lighting (46.2%). There was also a slight tendency for patients to experience VH more often at home than outdoors. However, there were also patients who hallucinated equally often at home and outdoors.

Patients were asked about their strategies for manipulating and resolving the hallucinations. Slightly more than a third of the patients were unable to intentionally manipulate the VH. About half of patients had different strategies for influencing their images. There was no clear tendency towards a particular technique. The disappearance of the VH, e.g. by blinking or closing the eyes was not possible in 30.8% of patients, while in another 30.8% the VH disappeared spontaneously without intentional action.

In general, patients communicated about their experiences. More than half (8/13) of the patients had spoken to someone from their personal surrounding about the hallucinations. Most frequently, patients contacted their family (30.8%). Overall, there was little concern that the images were caused by another (e.g., mental or neurological) condition. However, three patients (23.1%) had such concerns. In addition, at least six out of 13 patients (46.2%) had negative feelings when the hallucinations first occurred. Emotional evaluation of the images ranged from fear (2/13) or fear at the beginning (2/13) to discomfort (1/13), anger (1/13) or varied (1/13). The other patients were indifferent in the hallucinations (7/13). Three patients (23.1%) sought medical advice by consulting a physician, two ophthalmologists and one neurologist. Two of these patients seeking advice belonged to the group of worried patients who feared that they were suffering from another underlying disease. In all three cases, the physicians were able to diagnose CBS.

## Discussion

In the present study, a CBS prevalence of 26% was found in patients with a VA ≥ 0.5 logMAR. This confirms the range of previously reported prevalences of CBS in patients with low vision (6–34%). A recent meta-analysis found a prevalence of 19.7% among low-vision patients [26]. Furthermore, the prevalence of CBS in low-vision patients is comparable to other populations with various ophthalmic conditions, such as glaucoma patients (prevalence = 23%, *N* = 141 [12]), patients with retinal diseases (prevalence = 40%, *N* = 53 [8]; prevalence = 38%, *N* = 72 [27], prevalence = 39%, *N* = 1254 [28]) and in vision rehabilitation centres (prevalence = 35%, *N* = 225 [29]; prevalence = 24%, *N* = 50 [30]; prevalence = 19%, *N* = 2565 [15]). These findings suggest that low vision and ocular pathology are in fact risk factors for the development, as CBS occurred in all groups. Another factor might be the residual visual field. A previous study reported a correlation between VH and visual field recovery in patients undergoing vision restoration therapy (VRT) [31]. The study found that patients with large residual visual areas and diffuse visual field defects were more likely to experience VH during training stimuli. To date, only one study has investigated the occurrence of CBS in relation to the percentage of central and peripheral vision loss [32], and found a correlation between visual field and occurence of CBS. Reasons for different prevalences of existing studies could be due to both the varying definitions of CBS and different inclusion criteria. Sometimes researchers require their patients to have only complex hallucinations [9, 17] or exclude patients with hallucinations in other modalities [19, 20]. However, in a study investigating the association between visual and auditory hallucinations, no particularly high prevalence was found [18]. Furthermore, it might be possible that patients’ reluctance to talk about hallucinations, even when asked, varies from country to country due to cultural differences. For example, some patients may not want to report them for fear of being labelled as “mentally ill”, a genuine concern as some case reports show [23, 33, 34]. In one study, about one in ten patients attributed the hallucinations to mental illness [28].

Statistically, women were not significantly more often affected from CBS than men. However, our data suggest a preponderance of women among CBS patients, as also found in other studies [7, 8, 15, 23, 33, 35–44]. Mostly, it is assumed that the difference in sex distribution results from the fact that women tend to communicate more deliberately about unfamiliar events [15]. In a case report, Fernandes et al. observed a female patient who started to hallucinate only after the administration of oestrogens for osteoporosis therapy [45]. After discontinuation of the treatment, the VH disappeared. Maybe oestrogen could be a mediator in relation to the sex distribution in CBS. However, further studies are needed to confirm or disprove this theory.

Often, insight into the unreality of images is not gained immediately. 46.2% of patients did not initially recognise the images as unreal. Insight in the unreality of the hallucinations is a key feature that is often required for CBS diagnosis. De Morsier, who first described the syndrome in 1967, did not consider insight to be mandatory, as it can be subject to fluctuations [2]. Other commonly used diagnostic criteria such as those of Damas-Mora et al. [4] and Rabins [3] insist that insight, at least partial insight, is crucial for the diagnosis of CBS. In this context, it is important to note that the present study found that a considerable proportion of patients did not initially recognize the hallucinations as such, also recognised in the literature [9]. Yet, full insight is often an inclusion criterion, which may result in patients with delayed insight in the VH being excluded from some studies. There are few studies examining the onset for VH in relation to the onset of insight in them. Typically, delayed but then quickly gained insight was observed [11, 33, 34, 43, 46, 47].

Patterns, or so-called “simple” hallucinations, occurred just as frequently as other types of images such as people, body parts, or faces [18, 20, 33, 48–51]. In line with other studies [7, 40], the most frequent images were animals. The occurrence of simple hallucinations sometimes constitutes an exclusion of CBS. A variety of images are described as simple hallucinations, including flashes, sparks, bugs, or colour fields, snowflakes, mist, geometric shapes and patterns [33, 49]. However, simple hallucinations have been reported in up to 23% of patients who saw at least one other complex image [49]. The requirement that as least one complex image occurs alongside other, simpler hallucinations might be a useful tool to make an accurate differential diagnosis. Nevertheless, other studies have also observed the familiarity of simple hallucinations in CBS patients [11, 27, 28, 33, 40, 42, 48], with, for example, up to 63% of patients seeing patterns as a form of simple hallucinations in a 2014 survey by Cox and Ffytche [28].

Visual hallucinations occurred more frequently during the day (46.2%) and in bright surroundings (46.2%). In most studies, evening/night time [8, 20, 48, 52] and poor or dim lighting [7, 20, 40, 42, 48] were mentioned as favourable circumstances for the appearance of VH. In addition, it was observed in one case that improved lighting resolved the hallucinations [53], and in another study about one in five patients recognised the lighting intensity as potential trigger for VH [11]. These findings were not confirmed by the present study. However, it does not directly contradict the literature. A non-negligible proportion of patients describes bright or dazzling lights to trigger CBS hallucinations [7, 27, 40, 42]. Moreover, hallucinations may occur without any circadian affinity [48] or independently of ambient lighting [27]. Painter et al. showed that early visual cortex is hyperexcitable in CBS patients [54], suggesting that in an hyperexcitable cortex any stimulus, whether a poor visual input in the dark or a blurry but bright visual input in well-illuminated surroundings, can lead to overactivation of neurons. The brain would thereby create visual percepts with no correlation in the physical world, i.e. visual hallucinations. Although it may seem contradictory, these two clinical manifestations of CBS could be due to the same pathophysiology.

In addition, all patients experienced the hallucinations only with their eyes open. The hallucinations were generally (61.5%) not moving with the eyes. Literature shows that hallucinations with eyes open are quite common. In various studies, a large proportion of patients reported that their hallucinations occurred with their eyes open [11, 20, 48, 49, 52], spanning from 37.5[52]–97.9% [48]. Sometimes patients are also able to resolve their hallucinations by closing their eyes [33, 55, 56] suggesting that there is a close interaction between the brain and the eye in the development of the VH. The images do not arise spontaneously, but are triggered by an incomplete visual impression due to vision loss or visual field loss. The findings presented thus support the hypothesis of sensory deprivation (deafferentation) on the development of CBS hallucinations [57], emphasizing the importance of ocular pathology in the etiology of CBS. According to theory, ocular pathology with subsequently reduced visual input leads to spontaneous discharges in the visual cortex (hyperexcitability). As mentioned before, individuals with CBS do indeed have a hyperexcitable visual cortex [54]. The interaction between the diseased visual pathway and the brain is therefore crucial for the development of CBS. However, it should be noted that patients sometimes hallucinate with their eyes closed [58]. This interaction is also reflected in the fact that the visual hallucinations often do not move with the eyes, but generally stay in a certain place [20, 33]. However, this characteristic has only been investigated in a few studies. Sometimes the images themselves can also move across the visual field, such as marching soldiers [59]. Hallucinations seem to fit easily into the patient’s current spatial perception, implying again a close interaction between brain and visual pathway is underlying the aetiology of CBS and that spatial awareness is most likely unaffected by CBS.

38.5% of CBS sufferers did not communicate about their hallucinations. Most patients (76.9%) did not consult any physician. It has been observed in literature that many patients feel unwell because of their hallucinations, at least to some extent [20, 28, 33, 40, 48, 49]. Hallucinations can have a negative impact on the patients ‘ lives [20, 40], e.g. by interfering with daily activities [28]. It seems to be unusual for patients to talk about what they see. However, by talking about the hallucinations in the study setting, many of their patients seemed to feel relief. Nevertheless, not many studies have investigated whether patients turn to their social environment to talk about their experiences. In a study by Tan et al., three out of four patients spoke with their family about the images [51]. Teunisse et al. found that 73% of patients had not talked to their physician [20]. This was partly due to the fear of not taken seriously and partly due to not considering their problem important enough to address a doctor. One patient in the present study who did not consult his doctor was one of these who feared the VH might be caused by another underlying condition. It is possible that the patient avoided medical advice for fear of not being taken seriously or of being stigmatised as mentally ill. Future studies should investigate the psychosocial aspects of CBS in more detail.

The present study is limited by the small sample size of 50 patients, the interview design and the study setting. A larger sample size would provide more reliable results. However, other studies showed similar results with larger population sizes or deviated from the present results although examining similar population sizes. The interview was performed without a neuropsychiatric consultant having made any psychopathological findings. The cross-sectional study setting did not allow an evaluation of the course of the hallucinations over time. To monitor the development and progression, a longitudinal study would be more appropriate.

In summary, the present study assessed the prevalence of CBS in the population of low-vision patients and confirmed its clinical relevance. In addition, the characteristics of the visual hallucinations experienced by CBS-patients were described. Future studies should further address the effects of the residual visual field size on CBS. In addition, care must be taken during the selection process to ensure that the intake of psychoactive substances and hallucinogenic medication is confirmed by blood and urine samples alongside a thorough medical history and questioning. Moreover, using the Mini Mental State Examination (MMSE) and/or the Montreal Cognitive Assessment (MOCA) might be useful to screen for dementia. Furthermore, the management of CBS may benefit from encouraging patients to share their experiences and consult a physician. It may also be beneficial to conduct education campaigns for medical providers, as awareness of CBS among physicians appears to be low [60], but crucial to ensure that patients receive the appropriate support and care.

## Supplementary Information

Below is the link to the electronic supplementary material.

Supplementary file1 (DOCX 38 KB)
