# Supplementary material for: Epidemiology and phenomenology of the Charles Bonnet syndrome in low-vision patients
Source: Int Ophthalmol. 2024 Sep 10;44(1):375. doi: 10.1007/s10792-024-03298-0 (PMC11387450; doi:10.1007/s10792-024-03298-0)
Supplement: Supplementary file 1 — Supplementary file1 (DOCX 38 KB) [file 10792_2024_3298_MOESM1_ESM.docx]

Supplementary Information for the following manuscript

**Epidemiology and Phenomenology of the Charles Bonnet Syndrome in Low-Vision Patients**

*Authors*

Sophia E. G. Christoph^a,b^, Karl T. Boden^a^, Annette Pütz^a^, Kai Januschowski^a,b1^, Rudolf Siegel^c^, Berthold Seitz^d^, Peter Szurman^a,b^, André Schulz^a,b2^

*Affiliations*

^a^ Eye Clinic Sulzbach, Knappschaft Hospital Saar, Sulzbach/Saar, Germany

^b^ Klaus Heimann Eye Research Institute (KHERI), Sulzbach/Saar, Germany

^c^ Department of Psychology, Saarland University, Saarbrücken, Germany

^d^ Department of Ophthalmology, Saarland University Medical Center UKS, Homburg, Germany

^1^ Current address: Mount Saint Peter Eye Clinic, Trier, Germany

^2^ Current address: Rostock University Medical Center, Department of Ophthalmology, Rostock, Germany

*Corresponding Author*

Assistant Professor Dr. rer. nat. André Schulz; Address: Doberaner Str. 140, 18057 Rostock, Germany; Email: [andre.schulz@med.uni-rostock.de](mailto:andre.schulz@med.uni-rostock.de)

**Questionnaire for the study “Epidemiology and Phenomenology of the Charles Bonnet Syndrome in Low-Vision Patients”**

Patient's code number:

Visual acuity:

Right eye:

Left eye:

Eye diagnoses:

General Diagnoses:

Questions for the patient:

1. Have you ever perceived so called optical illusions?
2. When did you see these illusions for the first time?
3. Were you aware at the beginning that they were optical illusions?
4. How often do you perceive these mirages?
5. How long do the images last?
6. Which images do you see?
7. Do they match the environment?
8. Are the images in color or black and white?
9. Do the images move?
10. Do the illusions repeat themselves?
11. Do they look familiar to you?
12. Do you perceive the images with your eyes closed or open?
13. At what time of day do the images occur?
14. Do the images occur more in good or bad ambient lighting?
15. Do the images occur more when you are at home or outside the house?
16. Are you more likely to be alone or in company when the images occur?
17. Do the images tend to occur in certain situations?
18. Do the images move with your eye movements?
19. Are the images more distinct, equally distinct, or less distinct than real objects?
20. Have you told relatives or friends about these perceptions?
21. Have you ever talked to a doctor about the illusions?
22. What did the doctor tell you about it? Did he make a diagnosis? If so, which diagnosis?
23. Did the doctor advise you to further clarification or therapy? If so, which?
24. What feelings does the perception of the images trigger in you? At the beginning and currently?
25. Did you have or do you have the fear that the pictures are caused by a disease?
26. Can you influence the images at will?
27. Is it possible to make the images disappear? If so, how?
